# Acute Kidney Injury, Immune Thrombocytopenic Purpura, and the Infection That Binds Them Together: Disseminated Histoplasmosis

**DOI:** 10.1177/2324709617746193

**Published:** 2017-12-13

**Authors:** Pooja Sethi, Jennifer Treece, Chidinma Onweni, Vandana Pai, Sowminya Arikapudi, Lakshmi Kallur, Varun Kohli, Jonathan Moorman

**Affiliations:** 1 East Tennessee State University, Johnson City, TN, USA

**Keywords:** acute kidney injury, AKI, idiopathic thrombocytopenic purpura, ITP, disseminated histoplasmosis, human immunodeficiency virus, HIV

## Abstract

Untreated human immunodeficiency virus (HIV) can be complicated by opportunistic infections, including disseminated histoplasmosis (DH). Although endemic to portions of the United States and usually benign, DH can rarely act as an opportunistic infection in immunocompromised patients presenting with uncommon complications such as acute kidney injury and idiopathic thrombocytopenic purpura. We report a rare presentation of DH presenting with acute kidney injury and immune thrombocytopenic purpura in an immunocompromised patient with HIV.

## Introduction

The estimated incidence of histoplasmosis in adults aged 65 years and older in the United States is about 3.4 cases per 100 000 population.^1^ Histoplasmosis is a condition caused by *Histoplasma capsulatum*, which is a dimorphic fungus.^2^ In an immunocompetent host, histoplasmosis does not usually cause significant disease or illness, but in immunocompromised patients, severe illness can result, including disseminated histoplasmosis (DH). Common presentations of DH include chronic pulmonary disease, ocular manifestations, central nervous system involvement, and mediastinitis. DH can act as an opportunistic infection that can complicate untreated human immunodeficiency virus (HIV) presenting with rare complications such as acute kidney injury (AKI)^3^ and idiopathic thrombocytopenic purpura (ITP).^4^

Hemolytic uremic syndrome/thrombotic thrombocytopenic purpura (TTP) is more commonly implicated with concomitant thrombocytopenia and AKI. Several case reports exist regarding DH in patients with acquired immunodeficiency syndrome (AIDS) or HIV, but these reported cases do not present with both AKI and ITP.^5^

Other causes of ITP include infections such as *Helicobacter pylori*, viral illnesses (HIV, hepatitis), medication-induced (gold, quinine, β-lactam antibiotics), pregnancy, as well as autoimmune conditions (systemic lupus erythematosus).^6,7^

We report a rare presentation of DH presenting as AKI and ITP.

## Case Report

A 35-year-old African American female patient with a history of recently diagnosed, untreated HIV presented to the emergency department with a 2-day history of fever, vomiting, diarrhea, and fatigue. She denied taking any medications. On physical examination, she had a temperature of 103°F and was otherwise hemodynamically stable with normal blood pressure, heart rate, respiration rate, and pulse oximetry on room air. Her physical examination was notable for oral thrush and lower abdominal tenderness. Her cardiovascular and pulmonary examinations were benign. She did not have lymphadenopathy or hepatosplenomegaly. She did not have any petechia or ecchymoses on her skin, and there was no swelling of her lower extremities.

The patient’s urine analysis showed 53 white blood cells (WBCs) per high-power field and was leukocyte esterase positive but nitrite negative with no proteinuria. Her hemoglobin level was 8.0 g/dL, WBC count was 7000/µL with a normal differential, and platelet count was 220 000 platelets/µL. Her creatinine was 9.0 mg/dL, which was elevated from 1.2 mg/dL 2 weeks prior, and she had not been on any antibiotics or other medications between the 2 creatinine measurements. Her CD4 cell count was 19 cells/mm, and the viral load was 1 000 000 IU/mL.

**Figure 1.**
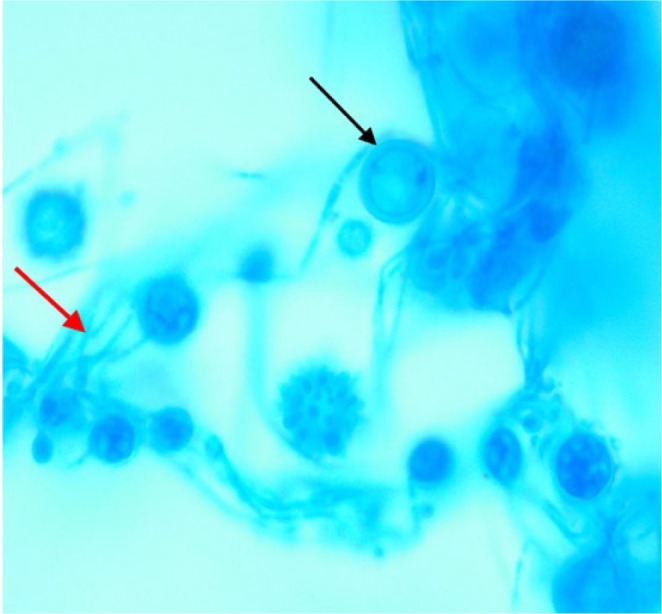
Thin tangled hyphae of *Histoplasma capsulatum* (red arrow) and several smooth walled macroconidia (black arrow).

**Figure 2.**
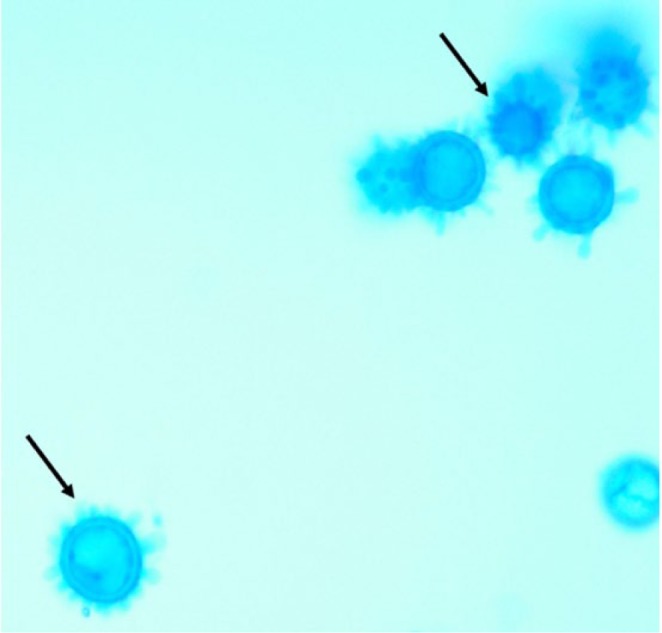
Trabeculate macroconidia with bumpy edges (black arrows) are diagnostic for *Histoplasma capsulatum.*

Despite treatment for pyelonephritis with intravenous (IV) ceftriaxone, the patient continued to be febrile and, at this time, her platelet count remained unchanged. Because she remained febrile, IV ceftriaxone was discontinued, and antibiotic coverage was broadened to IV vancomycin and piperacillin/tazobactam. On hospital day 3, the patient developed severe epistaxis, and her platelet count dropped to 26 000 platelets/µL. The patient had not received any heparin products since the start of her admission due to the patient being ambulatory and willing to wear a sequential compression device when lying in bed. The patient had no personal history or family history of easy bruising or easy bleeding. Blood gram stain showed intracellular yeast forms (Figures 1 and 2). The patient was started on IV amphotericin B for antifungal therapy to treat DH. Fungal culture and urine Histoplasma antigen confirmed the diagnosis of DH. Diagnosis of TTP was also a concern as the patient had a fever, anemia, thrombocytopenia, and renal failure. Though the patient’s ADAMTS13 level was elevated, favoring the diagnosis of TTP, the patient was ultimately diagnosed with ITP due to the absence of schistocytes on peripheral smear. High-dose intravenous corticosteroid administration resulted in a rapid improvement of her thrombocytopenia and confirmed the diagnosis of ITP. The patient was continued on a course of corticosteroids as well as amphotericin B. The patient’s AKI and thrombocytopenia continued to resolve over the course of a week, and she was discharged with a creatinine of 1.2 mg/dL and platelets count of 165 000 platelets/µL.

Unfortunately, the authors of this report no longer have access to this patient’s medical record and cannot comment further about the patient’s urine electrolytes (including fractional excretion of sodium), urine osmolarity, or more details about the creatinine and platelet levels relative to the timing of steroid initiation other than to point out that the creatinine level was elevated to 9.0 mg/dL at admission prior to corticosteroid, antibiotic, or antifungal therapy was initiated and returned to 1.2 mg/dL 4 days after initiation of corticosteroids and while receiving antibiotics and antifungal therapy. The platelet count was initially normal following IV ceftriaxone, IV vancomycin, and IV piperacillin/tazobactam therapy; decreased to 26 000 platelets/µL on hospital day 3 prior to the initiation of corticosteroids; and increased to 165 000 platelets/µL after receiving 4 days of corticosteroid therapy.

## Epidemiology

**Figure 3.**
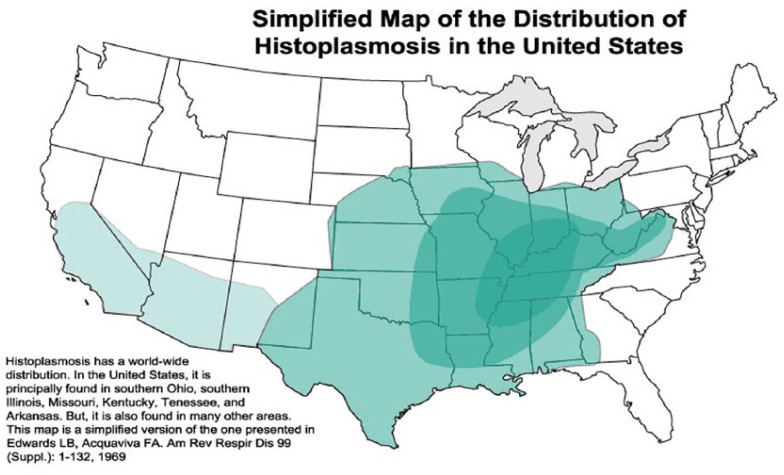
Endemic distribution of histoplasmosis in United States with the darker green areas having the highest incidence rates.^1^

Histoplasmosis is the most endemic mycosis in United State and is mainly distributed in the Midwest United States in the Mississippi-Ohio Valley as seen in Figure 3. The patient in this case was seen in the region where histoplasmosis is known to have a high incidence rate.

## Risk Factors

Patients with HIV that are not on antiviral therapy may have severely reduced CD4 counts, like the patient presented in this case. An impaired immune system, as seen in patients with low CD4 counts, causes these patients to be susceptible to opportunistic infections like DH. Patients may also be immunocompromised due to taking immunosuppressive medications like chemotherapy agents or chronic corticosteroids. Extremes in age can also predispose individuals to being immunocompromised and therefore susceptible to opportunistic infections like DH.

Patients in endemic areas may have a latent histoplasmosis infection. When patients who carry a latent histoplasmosis infection become immunosuppressed, they may develop DH due to their suppressed immune systems no longer being able to contain the infection. In nonendemic regions, the infection is most likely acutely acquired.^8^

Independent risk factors for patients with HIV to be susceptible for DH include acute renal failure, splenomegaly, respiratory insufficiency, hypotension, proteinuria, hepatomegaly, cutaneous lesions, and weight loss.^9^

## Clinical Features

Disseminated histoplasmosis can be nonspecific in presentation^2^ but can also have a multitude of presentations with mucocutaneous lesions, dyspnea or respiratory failure, hepatomegaly, splenomegaly, hematological derangements, and B symptoms like fever (in up to 95%),^9^ anorexia, and weight loss. Gastrointestinal symptoms are most common and are present in up to 70% of cases of DH.^9^ Our patient presented with fever, nausea, vomiting, fatigue, and hematologic abnormalities like anemia.

## Diagnosis

A high suspicion for DH should be kept in patients who have known immunocompromised state and present with nonspecific clinical features as described above. A CD4 count <75 is an indicator that the patient is in an immunocompromised state, which is the case in our patient. The unusual findings of an elevated ADAMTS13 raised the suspicion for a possible diagnosis of TTP. This arises from autoantibody-mediated inhibition of the ADAMST13 enzyme, but the patient did not have all 3 necessary criteria to meet the diagnosis of TTP. The 3 components of TTP include microangiopathic hemolytic anemia with hyperbilirubinemia, elevated lactate dehydrogenase (LDH), and schistocytes on the peripheral blood smear as well as fever and AKI. Although the last 2 criteria (fever and AKI) were met in this patient, the lack of the first criterion on blood smear excluded the diagnosis of TTP. Also, patients with TTP are generally more ill appearing than the patient in this case, and the improvement of the patient’s symptoms and labs on treatment of ITP with corticosteroids and antifungal treatment of DH supports the diagnosis of ITP over TTP. Therefore, following the exclusion of TTP, the patient was diagnosed with ITP, as ITP is a diagnosis of exclusion. Fungal culture and urine Histoplasma antigen confirmed the diagnosis of DH. Elevated LDH has been associated with hemophagocytosis syndrome and are also suggestive of DH in HIV patients.^9^

## Differential Diagnosis

HIV-associated nephropathy causes focal segmental glomerulosclerosis. Clinicians should be careful to exclude other causes of renal failure in HIV patients, particularly renal failure of acute onset like the patient presented in this case. Histoplasmosis causes renal insufficiency and shows granulomatous interstitial nephritis on kidney biopsy. Therapy with amphotericin B results in dramatic improvement in symptoms and lab values including rapid improvement in the creatinine level like the patient presented in this case. HIV-associated nephropathy, on the other hand, tends to be more indolent and progressive without a rapid improvement in kidney function on treatment. The diagnosis of TTP was on the differential diagnosis list as the patient had a fever, anemia, thrombocytopenia, and renal failure. The 3 components of TTP include microangiopathic hemolytic anemia with hyperbilirubinemia, elevated LDH, and schistocytes in the peripheral blood smear, fever, and AKI. Although the last 2 criteria are met, the first component is not completely satisfied, so the diagnosis is not TTP. Patients are generally more acutely ill with TTP. The improvement of the patient’s condition and overlapping features of DH in HIV and TTP favor ITP over TTP. Therefore, the patient in this case was diagnosed with ITP. Other differentials for thrombocytopenia include autoimmune diseases like antiphospholipid antibody syndrome, viral infections including hepatitis C and HIV, and certain medications.^10^

The presumed diagnosis of AKI was made in the patient presented in this case due to her not having any known renal pathology and with an abrupt increase in her creatinine level; her creatinine was 1.2 mg/dL 2 weeks before presenting to the hospital and was 9.0 mg/dL at presentation without the patient being exposed to any medications over this 2-week time period. The patient did not have proteinuria, which excludes the diagnosis of glomerulonephritis as the etiology of the worsening renal function. The authors of this case no longer have access to the patient’s medical records and therefore do not have the patient’s urine electrolyte or sediment measurements, so acute tubular necrosis remains a possible differential diagnosis as well as prerenal AKI. The patient did have sterile pyuria on her urinalysis with negative nitrite, so interstitial nephritis is also on the list of differential diagnoses. Reaction to a medication or infection are causes of interstitial nephritis, but the patient was not on any medications over the time that her creatinine increased from 1.2 mg/dL to 9.0 mg/dL and improved without the removal of an offending medication, which is the treatment of interstitial nephritis. The patient’s renal function improved with rehydration as well as antifungals and steroids. While Ogura et al described 2 cases of fungal acute glomerulointerstitial nephritis related to *Trichosporan laibachii* and *Candida albicans*, there have been case reports noting the association of histoplasmosis and interstitial nephritis.^11^ Given our patient’s concurrent findings, an isolated fungal induced acute interstitial nephritis is unlikely.

## Prognosis

Poor prognostic factors in patients with DH include dyspnea, platelet count <10 000/mm^3^, LDH greater than 2 times the upper limit of normal, hypotension, malnutrition with hypoalbuminemia, anemia, high levels of transaminases, acute renal failure, and respiratory insufficiency. Thrombocytopenia could be due to hemophagocytosis, disseminated intravascular coagulation, or bone marrow suppression. Patients with DH in the setting of HIV without concurrently taking antiviral therapy have a higher mortality rate than patients that are taking antiviral medications in the presence of an opportunistic infection. Early recognition of DH provides the opportunity for early targeted treatment.^9^

## Complications

Complications of DH include meningitis, acute respiratory distress syndrome, pericarditis, adrenal insufficiency, metabolic acidosis, and confusion as well as the rare complications of ITP and AKI. A delay in accurate diagnosis of the underlying etiology of the rapidly developing nephropathy can be fatal.^9^

## Pathogenesis of Complications

ITP causes direct platelet membrane damage or antibody-mediated platelet damage, releasing adenosine diphosphate and serotonin, which leads to platelet aggregation and increased clearance from circulation. AKI with Histoplasma antigen is caused by immune complexes collecting within the mesangium, which leads to granulomatous interstitial nephritis.^12^ Moreover, patients can be dehydrated, which furthers the injury to the kidneys.^9^

## Treatment

Disseminated histoplasmosis is treated with the antifungal agents including amphotericin B intravenously followed by oral itraconazole.^2^ The advantage of starting the therapy with amphotericin is that it is fungicidal and thus treats the fungemia, whereas the step-down drug that is preferred is oral itraconazole after the fungemia has resolved.^13^ ITP is treated with corticosteroids. HIV is treated with long-term therapy with antiviral medications, which allow the patient’s immune system to strengthen and ward off opportunistic infections as the CD4 count increases and viral load decreases. Amphotericin B results in an abrupt and significant improvement of kidney function and platelet count as was seen in the patient presented in this case.^13^

## Discussion

Histoplasmosis is the most common endemic mycosis in HIV patients and is of global distribution. The incidence of histoplasmosis can be lowered effectively by antiretroviral therapy in patients with HIV. Usually immunocompetent patients are asymptomatic, but immunosuppressed patients can present with acute pulmonary histoplasmosis, chronic pulmonary infection, and DH.^9^ DH should be considered in patients with advanced HIV with low CD4 counts who present with nonspecific symptoms, such as unexplained fever and weight loss. Ten percent to 20% of patients who present with DH have septic shock at presentation with fever, hypotension, coagulopathy, acute respiratory distress syndrome, as well as renal and hepatic failure.^9^ DH has a multitude of presentations with mucocutaneous, respiratory, gastrointestinal, or hematological derangements.^9^ There are very few case reports^3,14^ with complications of AKI and ITP in DH, and to our knowledge, no cases have been published with both complications of AKI and ITP in a single patient with DH, as was seen in the patient in this case report.

Compared with other opportunistic infections, DH presents in patients with HIV that have relatively lower CD4 counts. Other parameters like hemoglobin and platelet levels are also much lower in DH than with other opportunistic infections. Also, patients with advanced HIV who have other comorbid conditions like kidney failure, respiratory failure, and hepatomegaly are more prone to acquire DH.^9^ Thus, it is imperative to always consider the diagnosis of DH in HIV with such comorbidities. AKI is seen quite often with DH in HIV patients, and AKI acts as both a risk factor for and a sequela of DH. However, interestingly, in our patient, ITP was also present in the setting of AKI and DH. As the patient presented in this case had a creatinine of 9.0 mg/dL at presentation, which was prior to the initiation of amphotericin B or any antibiotics, her AKI was not secondary to medications. Patients with DH in other case reports also responded well to amphotericin B,^9^ confirming that amphotericin B is a good treatment option for DH. Our patient was not given any other antifungal medications prior to the manifestations of AKI and ITP. An alternative treatment to amphotericin B is itraconazole.^15^

## Conclusion

Untreated HIV can be complicated by opportunistic infections like DH. An objective of this article is to recognize DH as an opportunistic infection and understand its clinical manifestations. DH can present with rare complications like AKI and ITP. Another objective of this article is to discover the rare complications associated with DH and their appropriate management.
